# Outcomes following arthroscopic transosseous equivalent suture bridge double row rotator cuff repair: a prospective study and short-term results

**DOI:** 10.1051/sicotj/2015041

**Published:** 2016-02-15

**Authors:** Mohamed Abdelnabi Imam, Ashraf Abdelkafy

**Affiliations:** 1 The South West London Elective Orthopaedic Centre Dorking Road Epsom KT18 7EG London UK; 2 Orthopaedic Surgery and Trauma Department, Faculty of Medicine, Suez Canal University Circular Road 41522 Ismailia Egypt

**Keywords:** Transosseous equivalent, Suture bridge, Double row, Rotator cuff repair, Follow-up evaluation

## Abstract

*Background*: The transosseous-equivalent cross bridge double row (TESBDR) rotator cuff (RC) repair technique has been developed to optimize healing biology at a repaired RC tendon insertion. It has been shown in the laboratory to improve pressurized contact area and mean foot print pressure when compared with a double row anchor technique. Pressure has been shown to influence healing between tendon and bone, and the tendon compression vector provided by the transosseous-equivalent suture bridges may enhance healing. The purpose was to prospectively evaluate the outcomes of arthroscopic TESBDR RC repair.

*Methods*: Single center prospective case series study. Sixty-nine patients were selected to undergo arthroscopic TESBDR RC repair and were included in the current study. Primary outcome measures included the Oxford Shoulder Score (OSS), the University of California, Los Angeles (UCLA) score, the Constant-Murley (CM) Score and Range of motion (ROM). Secondary outcome measures included a Visual Analogue Scale (VAS) for pain, another VAS for patient satisfaction from the operative procedure, EuroQoL 5-Dimensions Questionnaire (EQ-5D) for quality of life assessment.

*Results*: At 24 months post-operative, average OSS score was 44, average UCLA score was 31, average CM score was 88, average forward flexion was 145°, average internal rotation was 35°, average external rotation was 79°, average abduction was 150°, average EQ-5D score was 0.73, average VAS for pain was 2.3, and average VAS for patient satisfaction was 9.2.

*Conclusion*: Arthroscopic TESBDR RC repair is a procedure with good post-operative functional outcome and low re-tear rate based on a short term follow-up.

## Introduction

Paradigm shifts in rotator cuff (RC) repair clearly occurred in the last two decades. This change was based on sound biomechanical principles, coupled with technological development of reliable and procedure-specific arthroscopic instruments [1].

However, there is a reported occurrence of re-tear in about 25%–40% of cases [2–4]. Re-tear is disappointing to both the surgeon and the patient. Efforts to prevent re-tears led to the introduction of the concept of footprint reconstruction which resulted in the use of double-row (DR) repair. Double-row RC repair techniques include medial and lateral rows of suture anchors in the repair construct. It provides a wider interface between the tendon and the original footprint of the humeral head [5–7]. However, recent literature review and meta-analysis revealed that the single-row (SR) repairs did not differ from the double-row repairs in functional outcome scores. Double-row repairs in comparison to single-row repairs revealed a trend toward lower radiographic proven re-tear rate, although the data did not reach statistical significance [8].

The transosseous-equivalent suture bridge RC repair technique has been developed to optimize healing biology at a repaired RC tendon insertion. There are several distinct advantages of the transosseous-equivalent technique. First, it has been shown in the laboratory to improve pressurized contact area and mean footprint pressure when compared with a double-row anchor technique. Pressure has been shown to influence healing between tendon and bone [9], and the tendon compression vector provided by the transosseous-equivalent suture bridges may enhance healing. Furthermore, the compressive nature of the suture bridges creates a low-profile repair that brings the medial mattress knots flush with the tendon, which may avoid tendon “edge instability” against the acromial-coracoacromial ligament arch [10].

The purpose of the current study was to evaluate the functional outcomes of transosseous-equivalent suture bridge double-row (TESBDR) arthroscopic RC repair.

Hypothesis generation was that TESBDR arthroscopic RC repair would show good functional outcome results.

## Methods

The current study was conducted as a single center prospective case series study.

Between April 2010 and July 2011, 69 patients were selected to undergo TESBDR arthroscopic RC repair and were included in the current study. All participants were screened for eligibility by the surgical team.

Inclusion criteria for patients selected to undergo the procedure were:

1. male and female patients;
2. symptomatic, MRI proven full-thickness RC tear;
3. full passive range of motion of the affected shoulder;
4. willingness to undergo standardized post-operative rehabilitation;
5. capacity to provide informed consent.

Exclusion criteria:

1. patients having a previous history of septic arthritis;
2. shoulder instability;
3. previous history of surgery of the affected shoulder;
4. patients diagnosed as having rheumatoid arthritis;
5. positive history of smoking [11] at the time when surgery was contemplated;
6. difficulty in communication due to cognitive impairment or poor language command;
7. massive, irreparable RC tears;
8. stiff shoulder;
9. rotator cuff tear arthropathy;
10. partial RC tears;
11. subscapularis tears;
12. fatty degeneration and atrophy of cuff muscles > 50%.

All participants were encouraged to remain in the study up to 24 months after surgery; however, participants were given the right to withdraw from the study at any time for any reason. The confidentiality of every patient was maintained at all times by allocating a number of each Case Report Form.

All patients were available for the follow-up evaluation 24 months post-operative.

Primary outcome measures included the Oxford Shoulder Score (OSS) [12], the University of California, Los Angeles (UCLA) score [13], the Constant-Murley (CM) Score [14], and Range of motion (ROM) [15].

Secondary outcome measures included a Visual Analogue Scale (VAS) for pain, another VAS for patient satisfaction from the operative procedure, and EuroQoL 5-Dimensions Questionnaire (EQ-5D) [16] for quality of life assessment. All patients were reviewed pre- and post-operatively by the first author.

Operative time, length of stay in hospital, pre-operative duration of symptoms, pattern and size of RC tear, and complications were recorded.

A trained member explained the study verbally to all participants. All foreseeable risks and potential benefits, which might occur, were discussed with all patients.

### Preoperative assessment

Once eligible patients were consented, the demographic characteristics and detailed medical history data were recorded. All scores were assessed and recorded preoperatively by the first author. The ROM was recorded using a standard goniometry. Forward flexion, internal rotation, and external rotation were measured in the supine position with the shoulder in 90 degrees of abduction in the scapular plane while the ipsilateral forearm was in neutral rotation [15, 17]. Shoulder abduction was also recorded post-operatively.

Pre-operative imaging included standard plain radiographs (anteroposterior in neutral, external, and internal rotation; and an axillary view) and MRI scans (1.5 T without gadolinium enhancement).

An experienced consultant specializing in musculoskeletal radiology reviewed all MR scans and assessed the size of each RC tear in the coronal and sagittal planes. The tear was categorized in the sagittal plane in three groups: tears < 1 cm, 1–3 cm, and 3–5 cm. The validity of the MRI measurements was assessed intra-operatively using the arthroscopic probe before debridement of the tear. The two methods matched.

### Surgical technique

All operations were performed with the patient in the lateral decubitus position under general anesthesia supplemented by an interscalene block. A standard shoulder arthroscopy instrument, a 30° arthroscope, and an arthroscopic pump set at 50 mm Hg of inflow pressure were used in all cases.

Diagnostic arthroscopy was performed through standard posterior viewing and anterior working portals, then the arthroscope redirected into the subacromial space. A lateral portal was also established. Excision of inflamed hypertrophied bursal tissue that might impede clearance of the space was then carried out, followed by subacromial decompression using a barrel burr (acromioplasty).

If needed, mobilization of the RC was accomplished by releasing the superior capsule off the superior labrum and the rotator interval from the supraspinatus tendon. The footprint on the greater tuberosity was debrided of soft tissue, thus exposing the underlying bone until bleeding surface. Typically, lateral portal (for instrumentation) and superior portal (for anchor placement) were used for RC repair. Not infrequently, however, the arthroscope had to be placed through an accessory posterolateral portal for better visualization of the RC, especially in bigger tears.

### Transosseous-equivalent double-row repair (see Figures 1–3)

A medial Biocorkscrew anchors^®^ (Arthrex, Naples, Florida) were placed first in the medial footprint and the FiberWire suture tails were passed through the tendon with the Multifire Scorpion Suture Passer^®^ (Arthrex, Naples, Florida). The medial row sutures were tied. Sutures were then passed over the lateral tendon with a BirdBeak suture passer^®^ (Arthrex, Naples, Florida) and were secured laterally with two Bioswivellock anchors^®^ (Arthrex, Naples, Florida). Repairs were performed with the shoulder abducted up to 30° to minimize tension on the repair.

**Figure 1.**
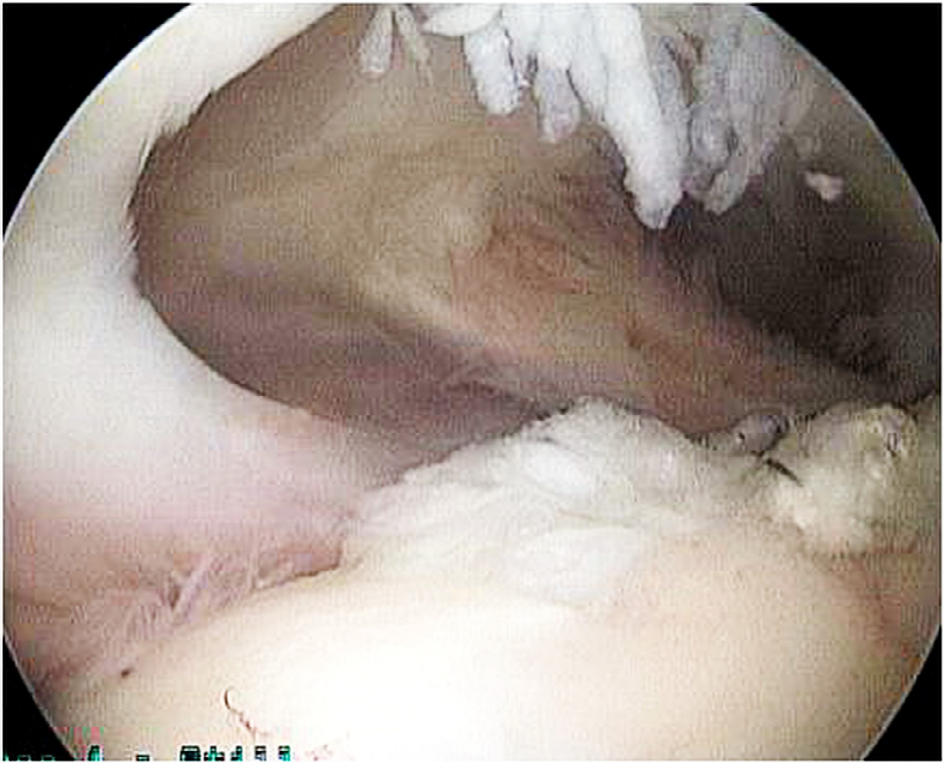
The supraspinatous tendon is frayed and inflamed.

**Figure 2.**
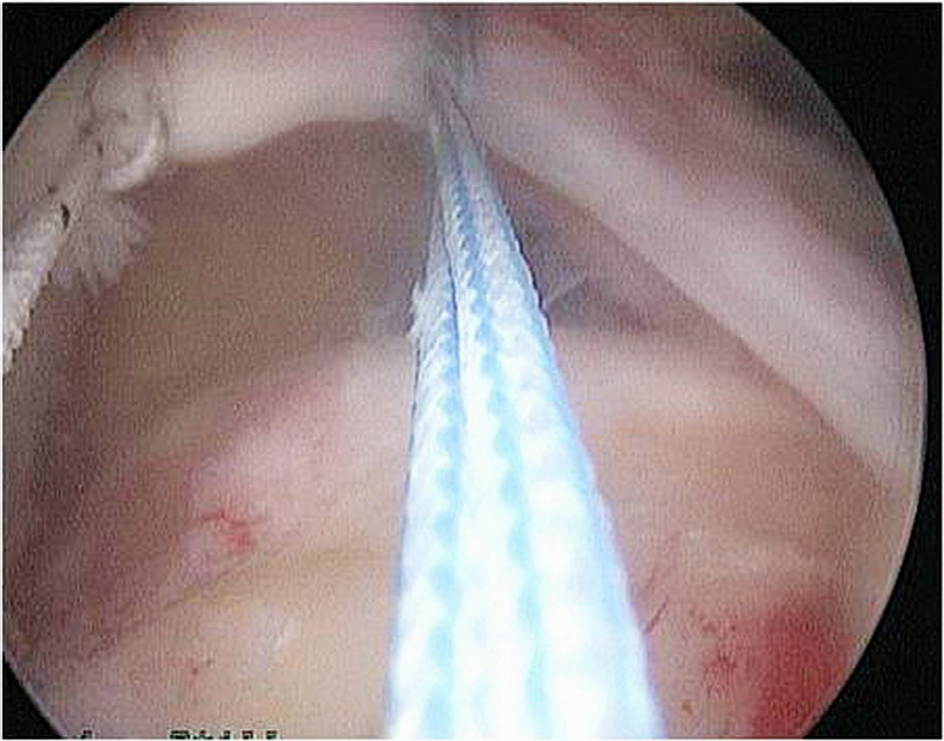
C-shaped, retracted, large size tear measuring 4 × 2.5 cm.

**Figure 3.**
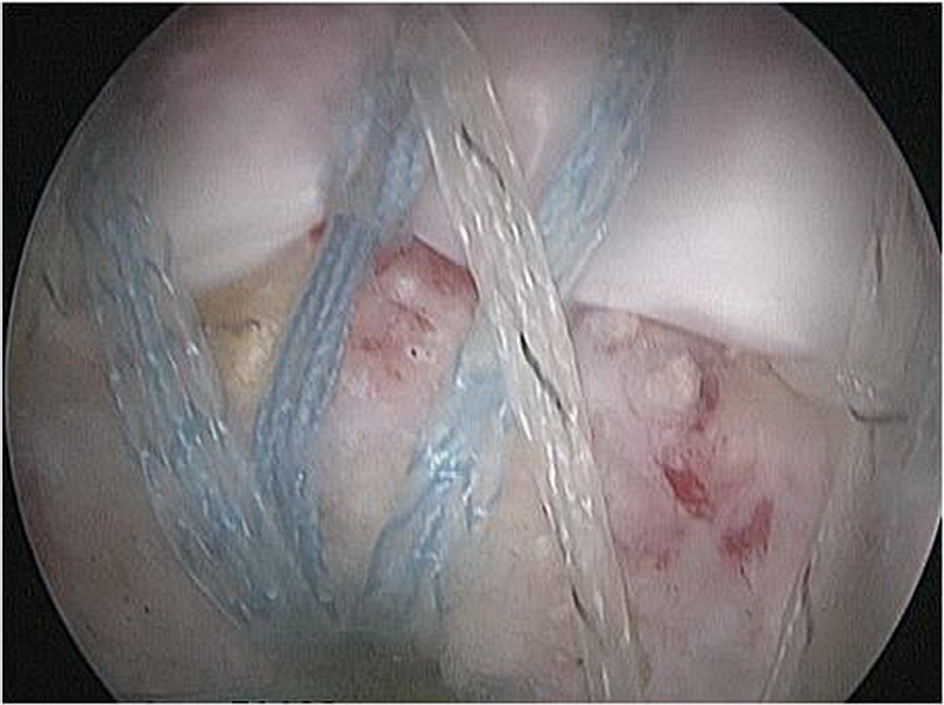
Arthroscopic transosseous-equivalent rotator cuff repair construct completed.

Tensioning of the FiberWire during second anchor insertion maximizes tendon compression and fixation of the tendon footprint on the tuberosity. A sliding arthroscopic knot is then tied over the recessed heads, locking the construct into place. Final repair is viewed and stability of the repair technique is checked.

### Post-operative care

#### Day of surgery

All patients were given information regarding the use of sling, activities of daily living, axillary hygiene, education in movements, and functional activities to be avoided. Advice regarding recovery of sensation from plexus nerve block if still active was also provided.

### Follow-up

Postoperatively, all patients used an abduction sling for four weeks and started on a rehabilitation program. Patients were seen every 2 weeks for the first 2 months and then once a month until the sixth month after surgery.

The following scores were used at 24 month post-operative: OSS, CM, UCLA, EQ-5D, VAS for pain, VAS for patient satisfaction in addition to the range of motion assessment.

No patients were lost for follow-up, and all completed the 24 month post-operative evaluation.

Complications were monitored and recorded continuously until 24 month post-surgery.

### Statistical analysis

Statistical analysis was performed using SPSS 16.0 for Windows. Comparisons were calculated using the Wilcoxon Signed Rank Test. *P*-value < 0.05 was considered statistically significant.

## Results

The average age of patients was 60.4 years (range 47–68). Average pre-operative duration of symptoms was 22 months (range 16–30). Average operative time was 120 min (range 100–130). Average post-operative hospital stay was 30 h (range 24–48).

All RC tears of patients included in the study were full-thickness tears. Measured intra-operatively using a graded arthroscopic probe, the mean size of tears was 2 × 1.5 cm (±0.5 × 0.5 cm). The smallest tear measured 0.5 cm × 1 cm, while the largest tear measured 4 cm × 3.5 cm. The tear shapes were principally U-shaped or crescent-shaped, while 2 tears were L-shaped. Sizes of the tears according to Bateman [18] classification were 41 Bateman grade II (1–3 cm), while 28 tears were Bateman grade III (3–5 cm).

At 24 months post-operative, primary outcome measures revealed that average OSS score was 44, average UCLA score was 31, average CM score was 88, average forward flexion was 145°, average internal rotation was 35°, average external rotation was 79°, and average abduction was 150° (see Tables 1 and 2). Secondary outcome measures revealed that average EQ-5D score was 0.73, average VAS for pain was 2.3, and average VAS for patient satisfaction was 9.2 (see Table 3).

**Table 1.** Primary outcome measures (shoulder scores) 24 months post-operative.

	Average	*SD*	Median	Mode	Range	Minimum	Maximum
OSS	44	8.5	40	41	6	39	45
UCLA score	31	6.1	29	30	8	27	35
CM score	88	9.7	86	86	15	80	96

*SD*: Standard deviation; OSS: Oxford Shoulder; UCLA: University of California, Los Angeles; CM: Constant-Murley.

**Table 2.** Primary outcome measures (ranges of motion) 24 months post-operative.

	Average	*SD*	Median	Mode	Range	Minimum	Maximum
Forward flexion	145	14.3	140	135	40	125	165
Internal rotation	35	8.3	30	32	11	28	39
External rotation	79	5.5	75	75	15	70	85
Abduction	150	12.1	142	135	30	125	155

*SD*: Standard deviation.

**Table 3.** Secondary outcome measures (visual analogue scales and quality of life score) 24 months post-operative.

	Average	*SD*	Median	Mode	Range	Minimum	Maximum
VAS for pain	2.3	0.4	2	2	3	0	3
VAS for patient satisfaction	9.2	1.1	8	8	3	7	10
EQ-5D score	0.73	0.02	0.74	0.72	0.08	0.7	0.78

*SD*: Standard deviation; VAS: visual analogue scale; EQ-5D: EuroQoL 5-Dimensions Questionnaire (a quality of life assessment score).

Comparing the results of the primary outcome measures pre-operative and at the time of follow-up evaluation (24 months post-surgery) showed significant improvement in average OSS score, average UCLA score, and average CM score (see Table 4).

**Table 4.** Comparison between pre-operative primary outcome measures (shoulder scores) and 24 months post-operative.

	Pre-operative	24 months post-operative	*P*-value
OSS (average)	23.5	44	<0.05
UCLA score (average)	14	31	<0.05
CM score (average)	45.5	88	<0.05

OSS: Oxford Shoulder; UCLA: University of California, Los Angeles; CM: Constant-Murley.

Comparing pre-operative range of motion and the range of motion at the time of follow-up evaluation, the average forward flexion, average internal rotation, and average external rotation improved significantly. However, average abduction did not improve significantly at the time of follow-up evaluation (see Table 5).

**Table 5.** Comparison between pre-operative primary outcome measures (ranges of motion) and 24 months post-operative.

	Pre-operative	24 months post-operative	Improvement	*P*-value
Forward flexion (average)	100°	145°	45°	<0.001
Internal rotation (average)	25°	35°	10°	<0.001
External rotation (average)	57°	79°	24°	<0.05
Abduction (average)	138°	150°	12°	>0.05

Comparing the pre-operative results of the secondary outcome measures and at the time of follow-up showed that average VAS for pain and average EQ-5D score improved significantly (see Table 6).

**Table 6.** Comparison between pre-operative secondary outcome measures (visual analogue scales and quality of life) and 24 months post-operative.

	Pre-operative	24 months post-operative	*P*-value
VAS for pain (average)	8.5	2.3	<0.05
EQ-5D (average)	0.41	0.73	<0.05

VAS: visual analogue scale; EQ-5D: EuroQoL 5-Dimensions Questionnaire (a quality of life assessment score).

Comparing the functional scores at the time of follow-up evaluation for patients with cuff tears Bateman III and those with Bateman II tears, the difference in average OSS score, average UCLA score, average CM score, average EQ-5D, average VAS for pain, and average VAS for patient satisfaction was not statistically significant (see Table 7).

**Table 7.** Comparison between Bateman III and II shoulder scores 24 months post-operative.

	Bateman III	Bateman II	*P*-value
OSS (average)	40	43	>0.05
UCLA score (average)	31	32	>0.05
CM score (average)	84	86	>0.05
EQ-5D score (average	0.76	0.74	>0.05
VAS for pain	2.1	2.2	>0.05
VAS for patient satisfaction	9.5	9.1	>0.05

Bateman: Classification of rotator cuff tear size; OSS: Oxford Shoulder; UCLA: University of California, Los Angeles; CM: Constant-Murley; EQ-5D: Quality of life score; VAS: Visual analogue scale.

Only one patient experienced superficial infection which was successfully treated with antibiotics.

## Discussion

The most noteworthy outcome of the current study is that TESBDR RC repair showed statistically significant improved functional scores at 24 months post-operative as average OSS score was 44, average UCLA score was 31, average CM score was 88, average forward flexion was 145°, average internal rotation was 35°, average external rotation was 79°, average abduction was 150°, average EQ-5D score was 0.73, average VAS for pain was 2.3, and average VAS for patient satisfaction was 9.2.

In an attempt to improve healing, rotator cuff repair techniques have evolved to create a stronger biomechanical construct. Double-row RC repair techniques added a row of suture anchor fixation lateral to the conventionally placed medial row that had been the standard fixation strategy for arthroscopic rotator cuff repairs. Biomechanical studies showed increased load to failure, improved contact areas and pressures, and decreased gap formation at the tendon-bone interface with double-row constructs [5, 7, 19, 20].

The anatomic benefit of double-row rotator cuff repairs was shown by Oguma et al. [21] as the potential for woven bone formation to anchor collagen fibers at the bone-tendon interface increases as the available contact area for the fibrovascular tissue interface increases. Although better than a single-row fixation, traditional double-row suture anchor repairs do not have the potential increased tendon-bone interface pressure [22].

In an effort to combine the stronger biomechanical repairs of the double-row construct with the increased tendon-bone interface pressure benefits, the transosseous-equivalent suture bridge repairs were developed [10].

Park et al. showed that the ultimate load to failure was significantly higher in the TESBDR repair than in the conventional double-row repair [23–25]. TESBDR configurations have been shown to maintain force contact over time better than both single- and double-row repairs [26]. In addition, many studies reported on the biomechanical superiority of TESBDR RC repair over the standard DR and single-row repair techniques due to the ability to provide compression through the footprint by increasing the contact area [10, 25, 27, 28]. This is achieved by connecting the medial and lateral rows, thus exerting compression throughout the repair, instead of only at the anchor insertion points.

Till now only few studies reported on the clinical outcome of TESBDR RC repair. Toussaint et al. [29] showed favorably comparable short-term results of clinical outcomes and structural integrity of TESBDR RC repair with those reported for other double-row suture anchor techniques employed in rotator cuff repairs. In a recent study, Park et al. [30] reported substantial improvements in pain and function after TESBDR RC repair but could not detect significant clinical difference between it and double-row RC repair. The results of the previous two studies match the results of the current study.

Park et al. [31] and Carbonel et al. [32] demonstrated significant improvement in functional outcome in patients who underwent double-row repair compared to those who underwent single-row repair, when used in patients with large to massive tears (≥3 cm). On the other hand, there was no difference between the repair techniques in patients with small to medium tears and this result matches the results of the current study.

Only two previous level I studies [33, 34] have examined patient satisfaction after arthroscopic RC repair. Both showed no statistically significant difference (*P* values; 0.986 and 0.3149) between double-row and single-row groups, with no statistically significant differences in the rate of return to work (*P* = 0.28).

Mihata et al. [35] documented 10.8%, 26.1%, and 4.7% re-tear rates, after the SR, DR, and compression DR techniques, respectively. From their study it is evident that the additional suture bridges decreased the re-tear rate.

Regarding complications, in the current study no re-tear symptoms as pain or weakness were reported until the time of the follow-up evaluation (24 months). Only one case of superficial infection was successfully treated with antibiotics. No other musculoskeletal complications, including neurological injuries, deep infections and anchor pull-outs, were reported.

### Points of strength of the current study

The strength of our study includes:

1. 100% follow-up;
2. the different shoulder functional scoring systems (OSS, UCLA, CM);
3. one score for quality of life assessment (EQ-5D);
4. two visual analogue scales (VAS for pain, VAS for patient satisfaction);
5. range of motion assessment.

### Limitations of the current study

(1) No follow-up MRI scans to assess the integrity of the RC repairs were used. This was not possible because of the associated high costs. (2) Short follow-up; however, as soft-tissue healing can be considered to be complete by 12 months [36], 24 months would be a sufficient follow-up period. (3) No re-tears occurred maybe due to the small number of cases (less than 100) included in the current study and the short-term follow-up. Re-tears if occurred would have been a point of interest to study and analyze in terms of the cause and how to prevent.

To conclude, arthroscopic transosseous-equivalent double-row rotator cuff repair is a safe procedure with good post-operative functional outcome based on a short-term follow-up.

## Conflict of interest

The authors declare no conflict of interest in relation with this paper.

## References

[1] Burkhart SS, Lo IK (2006) Arthroscopic rotator cuff repair. J Am Acad Orthop Surg 14(6), 333–346. 16757673 10.5435/00124635-200606000-00003

[2] Accousti KJ, Flatow EL (2007) Technical pearls on how to maximize healing of the rotator cuff. Instr Course Lect 56, 1–10. 17472287

[3] Cho NS, Lee BG, Rhee YG (2011) Arthroscopic rotator cuff repair using a suture bridge technique: is the repair integrity actually maintained? Am J Sports Med 39(10), 2108–2116. 21350064 10.1177/0363546510397171

[4] Lafosse L, Brozska R, Toussaint B, Gobezie R (2007) The outcome and structural integrity of arthroscopic rotator cuff repair with use of the double-row suture anchor technique. J Bone Joint Surg Am 89(7), 1533–1541. 17606793 10.2106/JBJS.F.00305

[5] Kim DH, Elattrache NS, Tibone JE et al. (2006) Biomechanical comparison of a single-row versus double-row suture anchor technique for rotator cuff repair. Am J Sports Med 34, 407–414. 16282581 10.1177/0363546505281238

[6] Ma CB, Comerford L, Wilson J, Puttlitz CM (2006) Biomechanical evaluation of arthroscopic rotator cuff repairs: double-row compared with single-row fixation. J Bone Joint Surg Am 88, 403–410. 16452754 10.2106/JBJS.D.02887

[7] Smith CD, Alexander S, Hill AM et al. (2006) A biomechanical comparison of single and double-row fixation in arthroscopic rotator cuff repair. J Bone Joint Surg Am 88, 2425–2431. 17079400 10.2106/JBJS.E.00697

[8] DeHaan A, Axelrad T, Kaye E, Silvestri L, Puskas B, Foster T (2012) Does double-row rotator cuff repair improve functional outcome of patients compared with single-row technique? A systematic review. Am J Sports Med 40, 1176–1185. 22156169 10.1177/0363546511428866

[9] Weiler A, Hoffmann RFG, Bail HJ, Rehm O, Sudkamp NP (2002) Tendon healing in a bone tunnel. Part II: Histologic analysis after biodegradable interference fit fixation in a model of anterior cruciate ligament reconstruction in sheep. Arthroscopy 18, 124–135. 11830805 10.1053/jars.2002.30657

[10] Park MC, Elattrache NS, Ahmad CS, Tibone JE (2006) “Transosseous-equivalent” rotator cuff repair technique. Arthroscopy 22(12), 1360.e1–1360.e5. 17157738 10.1016/j.arthro.2006.07.017

[11] Mallon WJ, Misamore G, Snead DS, Denton P (2004) The impact of preoperative smoking habits on the results of rotator cuff repair. J Shoulder Elbow Surg 13(2), 129–132. 14997086 10.1016/j.jse.2003.11.002

[12] Olley LM, Carr AJ (2008) The use of a patient-based questionnaire (the Oxford Shoulder Score) to assess outcome after rotator cuff repair. Ann R Coll Surg Engl 90(4), 326–331. 18492399 10.1308/003588408X285964 PMC2647197

[13] Kirkley A, Griffin S, Dainty K (2003) Scoring systems for the functional assessment of the shoulder. Arthroscopy 19(10), 1109–1120. 14673454 10.1016/j.arthro.2003.10.030

[14] Conboy VB, Morris RW, Kiss J et al. (1996) An evaluation of the Constant-Murley shoulder assessment. J Bone Joint Surg Br 78-B, 229–232. 8666631

[15] American Academy of Orthopaedic Surgeons (1965) Joint motion: method of measuring and recording. Chicago, IL, American Academy of Orthopaedic Surgeons.

[16] The EuroQol Group (1990) EuroQol-a new facility for the measurement of health-related quality of life. Health Policy 16(3), 199–208. 10109801 10.1016/0168-8510(90)90421-9

[17] Franceschi F, Ruzzini L, Longo UG, Martina FM, Zobel BB, Maffulli N, Denaro V (2007) Equivalent clinical results of arthroscopic single-row and double-row suture anchor repair for rotator cuff tears: a randomized controlled trial. Am J Sports Med 35(8), 1254–1260. 17554104 10.1177/0363546507302218

[18] Habermeyer P, Magosch P (2006) Classifications of complete rotator cuff tears according to Bateman, in Classifications and Scores of the Shoulder. Springer, Berlin, Heidelberg, p. 23.

[19] Meier SW, Meier JD (2006) The effect of double-row fixation on initial repair strength in rotator cuff repair: a biomechanical study. Arthroscopy 22, 1168–1173. 17084292 10.1016/j.arthro.2006.07.004

[20] Meier SW, Meier JD (2006) Rotator cuff repair: the effect of double-row fixation on three-dimensional repair site. J Shoulder Elbow Surg 15, 691–696. 17126241 10.1016/j.jse.2006.03.004

[21] Oguma H, Murakami G, Takahashi-Iwanaga H et al. (2001) Early anchoring collagen fibers at the bone-tendon interface are conducted by woven bone formation: light microscope and scanning electron microscope observation using a canine model. J Orthop Res 19, 873–880. 11562136 10.1016/S0736-0266(01)00021-3

[22] Dines JS, Bedi A, ElAttrache NS et al. (2010) Single-row versus double-row rotator cuff repair: techniques and outcomes. J Am Acad Orthop Surg 18, 83–93. 20118325 10.5435/00124635-201002000-00003

[23] Park MC, Idjadi JA, Elattrache NS et al. (2008) The effect of dynamic external rotation comparing 2 footprint-restoring rotator cuff repair techniques. Am J Sports Med 36, 893–900. 18272799 10.1177/0363546507313092

[24] Park MC, ElAttrache NS, Tibone JE et al. (2007) Part I: Footprint contact characteristics for a transosseous-equivalent rotator cuff repair technique compared with a double-row repair technique. J Shoulder Elbow Surg 16, 461–468. 17321161 10.1016/j.jse.2006.09.010

[25] Park MC, Tibone JE, ElAttrache NS et al. (2007) Part II: Biomechanical assessment for a footprint-restoring transosseous-equivalent rotator cuff repair technique compared with a double-row repair technique. J Shoulder Elbow Surg 16, 469–476. 17321158 10.1016/j.jse.2006.09.011

[26] Mazzocca AD, Bollier MJ, Ciminiello AM et al. (2010) Biomechanical evaluation of arthroscopic rotator cuff repairs over time. Arthroscopy 26, 592–599. 20434655 10.1016/j.arthro.2010.02.009

[27] Burkhart SS, Cole BJ (2010) Bridging self-reinforcing double-row rotator cuff repair: we really are doing better. Arthroscopy 26(5), 677–680. 20434667 10.1016/j.arthro.2010.02.007

[28] Apreleva M, Ozbaydar M, Fitzgibbons PG, Warner JJP (2002) Rotator cuff tears: the effect of the reconstruction method on three-dimensional repair site area. Arthroscopy 18, 519–526. 11987064 10.1053/jars.2002.32930

[29] Toussaint B, Schnaser E, Bosley J, Lefebvre Y, Gobezie R (2011) Early structural and functional outcomes for arthroscopic double-row transosseous-equivalent rotator cuff repair. Am J Sports Med 39(6), 1217–1225. 21427446 10.1177/0363546510397725

[30] Park JY, Lee SY, Chung SW, Zulkifli H, Cho JH, Oh KS (2013) Clinical comparison between double-row and transosseous-equivalent repairs for medium to large size rotator cuff tears. Arch Orthop Trauma Surg 133(12), 1727–1734. 24158719 10.1007/s00402-013-1872-9

[31] Park JY, Lhee SH, Choi JH et al. (2008) Comparison of the clinical outcomes of single- and double-row repairs in rotator cuff tears. Am J Sports Med 36, 1310–1316. 18413680 10.1177/0363546508315039

[32] Carbonel I, Martinez AA, Calvo A, Ripalda J, Herrera A (2012) Single-row versus double-row arthroscopic repair in the treatment of rotator cuff tears: a prospective randomized clinical study. Int Orthop 36(9), 1877–1883. 22584619 10.1007/s00264-012-1559-9 PMC3427450

[33] Charousset C, Grimberg J, Duranthon LD, Bellaiche L, Petrover D (2007) Can a double-row anchorage technique improve tendon healing in arthroscopic rotator cuff repair? Am J Sports Med 35, 1247–1253. 17452513 10.1177/0363546507301661

[34] Koh KH, Kang KC, Lim TK, Shon MS, Yoo JC (2011) Prospective randomized clinical trial of single- versus double-row suture anchor repair in 2- to 4-cm rotator cuff tears: clinical and magnetic resonance imaging results. Arthroscopy 27(4), 453–462. 21444007 10.1016/j.arthro.2010.11.059

[35] Mihata T, Watanabe C, Fukunishi K, Ohue M, Tsujimura T, Fujiwara K, Kinoshita M (2011) Functional and structural outcomes of single-row versus double-row versus combined double-row and suture-bridge repair for rotator cuff tears. Am J Sports Med 39(10), 2091–2098. 21785001 10.1177/0363546511415660

[36] Nho SJ, Adler RS, Tomlinson DP, Allen AA, Cordasco FA, Warren RF, Altchek DW, MacGillivray JD (2009) Arthroscopic rotator cuff repair: prospective evaluation with sequential ultrasonography. Am J Sports Med 37(10), 1938–1945. 19531660 10.1177/0363546509335764

